# Development of an Indirect ELISA Kit for Rapid Detection of Varicella-Zoster Virus Antibody by Glycoprotein E

**DOI:** 10.3389/fmicb.2022.897752

**Published:** 2022-04-27

**Authors:** Yan Niu, Aiping Wang, Jingming Zhou, Hongliang Liu, Yumei Chen, Peiyang Ding, Yanhua Qi, Chao Liang, Xifang Zhu, Gaiping Zhang

**Affiliations:** ^1^School of Life Sciences, Zhengzhou University, Zhengzhou, China; ^2^Henan Longhu Modern Immunity Laboratory, Zhengzhou University, Zhengzhou, China; ^3^College of Agriculture, Peking University, Beijing, China

**Keywords:** varicella-zoster virus, glycoprotein E, CHO cells, indirect ELISA kit, antibodies detection

## Abstract

Varicella-zoster virus (VZV), a highly infectious agent that causes varicella (chickenpox), can also cause zoster (shingles), a disorder that is frequently associated with severe neuralgia. A reliable serological VZV diagnostic assay would be useful for identifying unprotected individuals and for surveilling post-vaccination immunoprotection status. Toward this goal, VZV membrane glycoprotein E (gE), the immunodominant VZV protein, served as target antigen in an indirect ELISA kit developed here to detect anti-VZV antibodies in clinical samples. For target antigen preparation, Chinese hamster ovary (CHO) cells were modified to express and secrete the VZV gE ectodomain, which was subsequently purified and used as coating antigen in an indirect ELISA. Ultimately, the optimal purified gE coating antigen concentration was determined to be 2 μg.ml^−1^ and the OD_450nm_ detection cutoff value was 0.286. The coefficient of variation (CV) of intra-assay and inter-assay were <10 and 15%, respectively. A comparative test of 66 clinical samples showed that the coincidence rate was 93.9% between the indirect ELISA and a commercial varicella-zoster virus IgG ELISA kit. Thus, the indirect ELISA kit developed here may be useful for achieving rapid, sensitive, and specific detection of anti-VZV antibodies.

## Introduction

Varicella-zoster virus (VZV) is a human-specific virus and infection does not occur, or is highly restricted, in other species (Zerboni et al., 2014). It usually causes varicella (chickenpox) in children and zoster (shingles) in adult. Primary infection with VZV results in varicella, which is generally a mild, self-limiting disease in healthy children and typically presents as vesicles. However, in immunocompromised patients and susceptible individuals (particularly pregnant women), the infection of VZV can be more serious and even life-threatening (Harger et al., 2002; Tunbridge et al., 2008; Charlier et al., 2014). After primary infection, the virus establishes permanent latency in the cranial nerves and dorsal root ganglia. Reactivation of VZV occurs later in life, leading to herpes zoster (HZ), commonly manifesting as a painful, unilateral, vesicular, and dermatomal rash that typically heals in several weeks. A common complication of HZ is post-herpetic neuralgia (PHN), which manifests as a chronic pain disorder with increased incidence in the elderly (Drolet et al., 2010; Ilyas and Chandrasekar, 2020).

Varicella-zoster virus is the member of *alpha Herpesviridae* family, genus *Varicellovirus*. Five phylogenetic VZV clades have been identified, but the most disparate still have 99.8% sequence conservation (Breuer, 2010; Zerboni et al., 2014). VZV virion is 80 ~ 120 nm in diameter and made up of genome, icosahedral capsid core, tegument layer, and lipid-rich envelope (composed of at least nine viral glycoproteins). The VZV genome is a linear double-stranded DNA molecule of ~125 kb that encodes at least 71 known or predicted open reading frames (ORFs; Davison and Scott, 1986; Quinlivan and Breuer, 2006; Cohen, 2010; Zerboni et al., 2014). VZV envelope glycoprotein E (gE) is a typical type I membrane protein (623 amino acid) encoded by ORF68 and considered the major protective antigen (Gershon and Gershon, 2010; Dendouga et al., 2012). It is a multifunctional protein important for viral replication, cell-to-cell transmission, envelopment, and possibly entry (Berarducci et al., 2006).

A reliable, generally available, and highly sensitive serological test for VZV-specific antibody would be helpful for (a) quickly evaluating the serological status of patients and hospital personnel when nosocomial varicella breaks out (Demmler et al., 1988); (b) evaluating the immune status of immunocompromised individuals, pregnant women, and healthcare workers (Sauerbrei and Wutzler, 2006); (c) evaluating the efficacy of varicella vaccine or HZ vaccine; (d) performing seroepidemiological survey; and (e) screening VZV-seronegative candidates for varicella vaccine (Baracco et al., 2015).

A variety of methods for the antibody detection to VZV have been described. Fluorescent antibody to membrane antigen assay (FAMA) is an accepted gold standard assay for the indicator of immune status to VZV (Maple et al., 2016). However, it is technically complex, time-consuming, subjective, and unsuitable for testing large numbers of sera. The ELISA has the advantages of high sensitivity, simplicity, time saving, and high throughput. The gpELISA coated with purified VZV glycoprotein (gp) was developed by Merck Sharp & Dohme Research Laboratories, and it has been used to measure the seroconversion rate in vaccinated clinical trials (Wasmuth and Miller, 1990; Hammond et al., 2006; Maple et al., 2006).

The main objects of FAMA assay and gpELISA are the specific VZV antibodies to envelope glycoproteins, and the gE protein is the major and most immunogenic glycoprotein of VZV. Therefore, CHO cells were used to express VZV gE ectodomain in this study. Then, we developed an indirect ELISA kit coated with gE protein for rapid detection of VZV antibodies. It might can be used for the auxiliary diagnosis of VZV infection, vaccine efficacy studies, and seroepidemiological surveillance activities.

## Materials and Methods

### Cells, Plasmids, and Reagents

DH5α competent cells were purchased from Genscript (Nanjing, China). Human embryonic kidney 293T (HEK293T) cells were obtained from ATCC (Manassas, VA, United States) and cultured with Dulbecco’s modified Eagle’s medium (Solarbio, Beijing, China) with 10% (v/v) fetal bovine serum (FBS, Gibco-BRL, United States). CHO cells were obtained from Invitrogen (CA, United States) and maintained with SMM CHO-SI medium (serum-free, supplemented with 4 mM L-glutamine, Beijing Sino Biological company). The pLVX-IRES-ZsGreen1, pMD2.G, and psPAX2 plasmids were stored in our laboratory. The anti-VZV positive and negative sera were purchased from National Institutes for Food and Drug Control of China. The clinical serum samples were from healthy volunteers. All chemical reagents used in the experiments were obtained from commercial sources and of analytical grade.

### Construction of pLVX-gE-IRES-ZsGreen1

The optimized VZV gE protein gene (GenBank No. MF898328.1) according to the codon bias of CHO was synthesized by Sangon Biotech Co., Ltd (Shanghai, China). A pair of primers (gE-*EcoRI*-F, 5′-CCGGAATTCATGGGAACAGTGAATAAGCCTGTGGTGGGC-3′ and gE-XhoI-R, 5′-CCGCTCGAGTCAGGCCAGGCCGCCGGTCCAAG-3′) was designed to amplify the VZV gE ectodomain gene (1–1,638 bp). Then the gene was cloned into the pLVX-IRES-ZsGreen1 lentiviral expression vector to construct the recombinant expression plasmid pLVX-gE-IRES-ZsGreen1. The connection product was transformed into DH5α competent cells, and the single clone was selected from the LB culture plate containing ampicillin. The pLVX-gE-IRES-ZsGreen1 recombinant plasmid was extracted and further confirmed to be constructed correctly *via* digestion with restriction enzymes *EcoRI* (NEB) and *XhoI* (NEB) followed by DNA sequence analysis.

### Generation of CHO-gE Cell

HEK293T cells were used to produce pseudotyped lentivirus particles. First, three plasmids, pLVX-gE-IRES-ZsGreen1 (1.45 μg), psPAX2 (2.94 μg), and pMD2.G (1.61 μg), were mixed with 12 μl jet PRIME® (Polyplus-transfection® SA, Illkirch, France) and subsequently incubated in 200 μl jet PRIME® buffer for 10 min at room temperature. Then, the mixture was added dropwise to HEK293T cells. The fluorescence was observed at 24 and 48 h post-transfection under the inverted fluorescence microscope (Olympus), respectively. Finally, the supernatant containing the pLVX-gE-IRES-ZsGreen1 lentivirus was collected.

Chinese hamster ovary cells were transduced with the pLVX-gE-IRES-ZsGreen1 lentivirus according to methods described previously (Du and Tikoo, 2010). Briefly, CHO cells (1 × 10^5^ cells/well) in 12-well plates were incubated with lentivirus (1:1, v/v) diluted in SMM CHO-SI Medium. The fluorescence was observed at 72 h post-transduction and the cells were subcloned *via* a limiting dilution method in 96-well cell plates. Finally, the CHO-gE cell with highest expression of gE was selected by sandwich ELISA. Briefly, 96-well ELISA plates (MaxiSorp, Thermo Fisher Scientific Inc., United States) were pre-coated with commercial gE mAb (Abcam, 2 μg.ml^−1^, 100 μl/well) diluted in 0.05 M carbonate–bicarbonate buffer (CBS, pH 9.6) and incubated at 37°C for 2 h. After washing with PBST (PBS containing 0.05% Tween-20), the plates were saturated with 200 μl of PBST containing 5% skim milk at 37°C for 2 h. Then, 100 μl of the cell supernatant was added to each well and incubated at 37°C for 1 h. After washing, 100 μl/well of anti-VZV positive serum (1: 500 dilution in PBS) was incubated at 37°C for 1 h. Then the wells were washed again, 100 μl/well of HRP-conjugated goat anti-human IgG (H + L; Proteintech, Wuhan, China) diluted in PBST containing 5% skim milk was incubated at 37°C for 1 h. After washing, the reactions were developed using 3,3′,5,5′-tetramethylbenzidine (TMB) for 5 min and stopped by 2 M H_2_SO_4_. Finally, the OD_450_ nm was measured by a plate reader (Bio-Rad).

### Expressing and Purification of gE Protein

To prepare truncated gE protein, the selected CHO-gE cell was shaking cultured for 12 ~ 14 days and fed with 1.5% SMS CHO-GS-SUPI cell culture supplement (Beijing Sino Biological company) every day. After centrifugation with 500 *g* for 10 min, the gE protein in the supernatant was collected and subsequently purified by Q-Sepharose® Fast Flow column described previously (Haumont et al., 1996). The purified protein was analyzed by SDS-PAGE and western blotting. The protein concentration was measured by a BCA protein assay kit (Solarbio, Beijing, China).

### Western Blot Analysis

Purified gE protein was resolved by 12% SDS-PAGE gels and transferred to PVDF membrane. The PVDF membrane was saturated with 5% skimmed milk at 4°C overnight. Then, the membrane was incubated with the anti-VZV positive serum (1: 500 dilution in PBS) at 37°C for 1 h. After washing with PBST three times, the membrane was incubated with HRP-conjugated goat anti-human IgG (H + L; 1: 5,000 dilution in 5% skimmed milk) at 37°C for 1 h. After washing three times, the blots were developed by AEC (ZSGB-BIO, Beijing, China).

### Development of Indirect ELISA Kit

Indirect ELISA kit was developed for rapid detection of varicella-zoster virus antibodies. In the indirect ELISA, optimal dilutions of antigen, serum, and HRP-conjugated goat anti-human secondary antibody were studied by the checkerboarding. The optimal indirect ELISA was as follows. Around 96-well ELISA plates (MaxiSorp, Thermo Fisher Scientific Inc., United States) were coated with gE protein (2 μg.ml^−1^, 100 μl/well) diluted in 0.05 M carbonate–bicarbonate buffer (CBS, pH 9.6) and incubated at 37°C for 2 h. Plates were washed with PBST three times and saturated with 5% skim milk (200 μl/well) at 4°C overnight. After washing three times, the plates were incubated with the clinical serum samples (1: 100 dilution in the PBS, 100 μl/well) at 37°C for 30 min. After washing seven times, the wells were added with HRP-conjugated goat anti-human secondary antibody (1: 5,000 dilution in 5% skimmed milk, 100 μl/well) and incubated at 37°C for 30 min. After washing seven times, the reactions were developed using tetramethylbenzidine (TMB) (100 μl/well) for 5 min and stopped with 2 M sulfuric acid (100 μl/well). The absorbance was measured immediately at 450 nm on a microplate reader. When the OD_450_ nm was >0.286, the sample was positive. Otherwise, the sample was negative.

### Detection of Clinical Serum Samples

To evaluate the performance of established indirect ELISA in serum detection, a comparison experiment was performed. Around 66 clinical samples were analyzed for the presence of VZV antibodies using the indirect ELISA kit developed in our study and a commercial varicella-zoster virus IgG ELISA kit (Virion-Serion, Germany), separately.

## Results

### Construction of pLVX-gE-IRES-ZsGreen1

**Figure 1:**
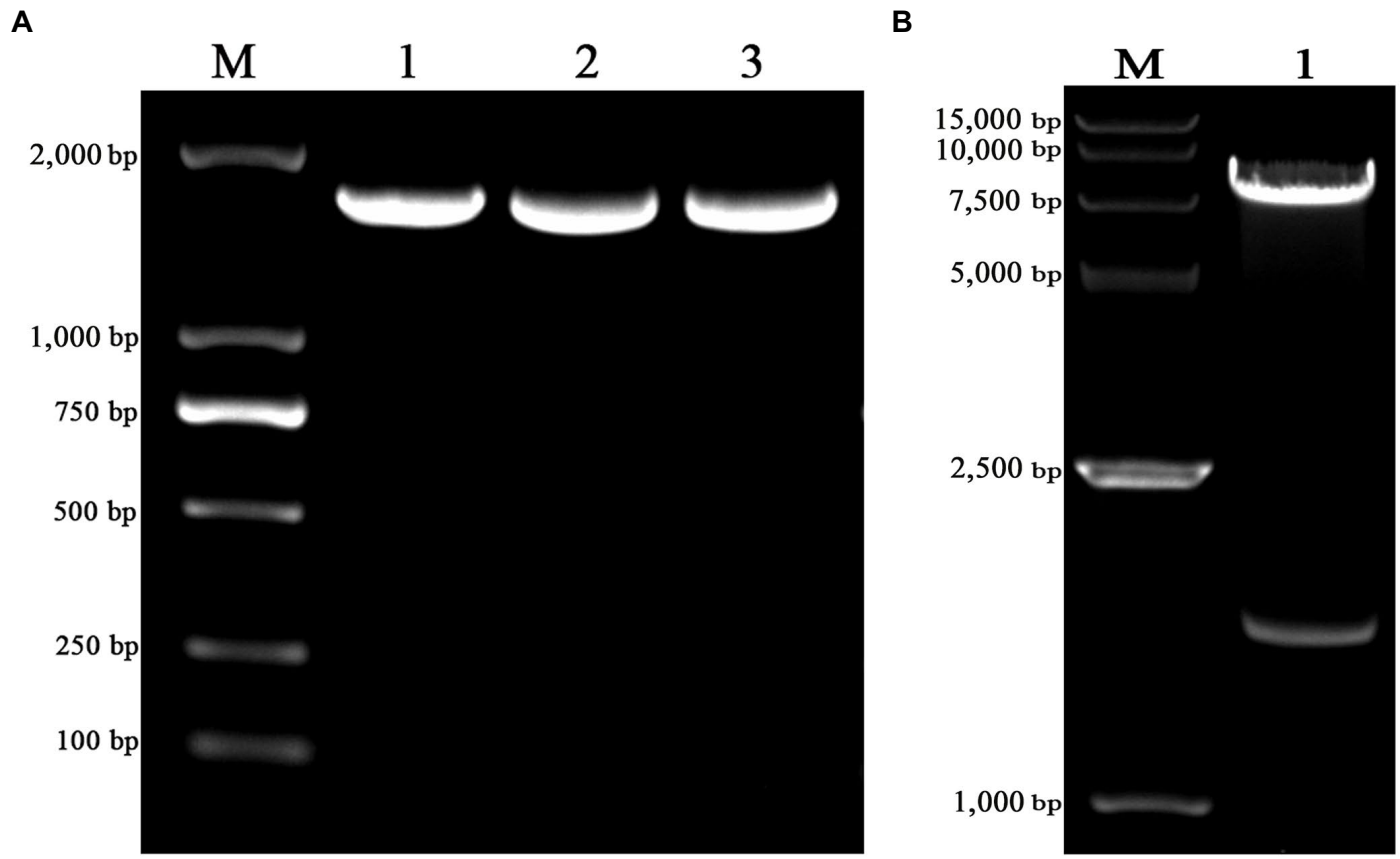
The construction of pLVX-gE-IRES-ZsGreen1 recombinant plasmid. **(A)** The PCR product of varicella-zoster virus (VZV) glycoprotein E (gE) ectodomain gene using the primers gE-*EcoRI*-F and gE-XhoI-R. **(B)** The enzyme identification results of pLVX-gE-IRES-ZsGreen1 recombinant plasmid with *EcoRI* and *XhoI*.

The truncated VZV gE gene was amplified with the length of 1,656 bp (Figure 1A) and cloned into the pLVX-IRES-ZsGreen1 lentiviral expression vector with restriction enzyme *EcoRI* and *XhoI*. The pLVX-gE-IRES-ZsGreen1 recombinant plasmid was extracted from DH5α cells and the sequencing result was correct. As shown in Figure 1B, the enzyme identification results showed that the recombinant plasmid was successfully digested into two fractions (1,648 bp and 8,196 bp) with *EcoRI* and *XhoI*. The results indicated that we successfully constructed the pLVX-gE-IRES-ZsGreen1 recombinant plasmid.

### Generation of CHO-gE Cell

**Figure 2:**
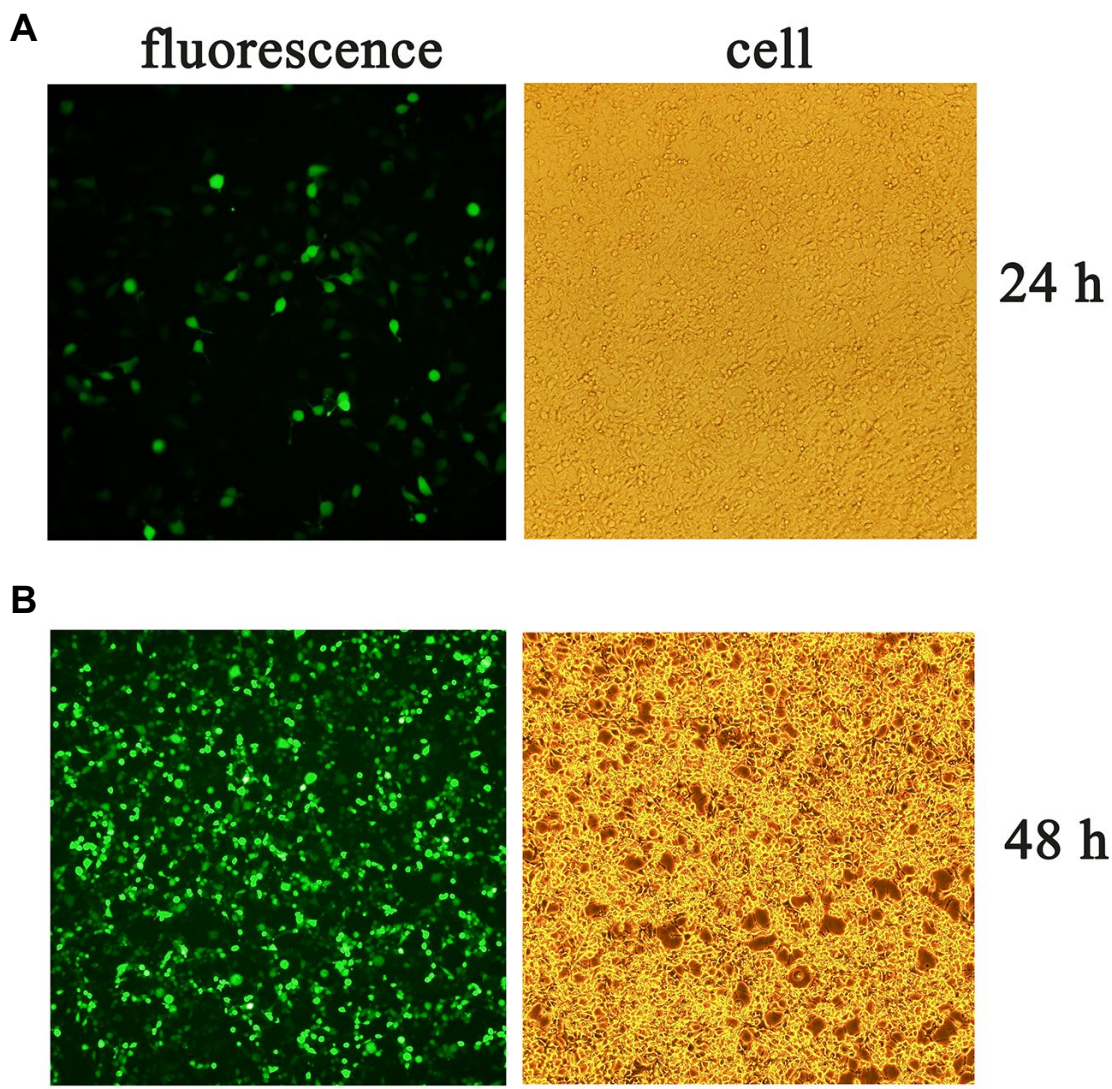
The fluorescence and cells at 24 **(A)** and 48 h **(B)** post-transfection.

Three plasmids pLVX-gE-IRES-ZsGreen1, psPAX2, and pMD2.G were co-transfected into 293T cells to obtain lentivirus. The fluorescence and cells were observed at 24 (Figure 2A) and 48 h (Figure 2B) post-transfection, respectively. Then, the supernatant containing the pLVX-gE-IRES-ZsGreen1 lentivirus was collected at 48 h post-transfection.

**Figure 3:**
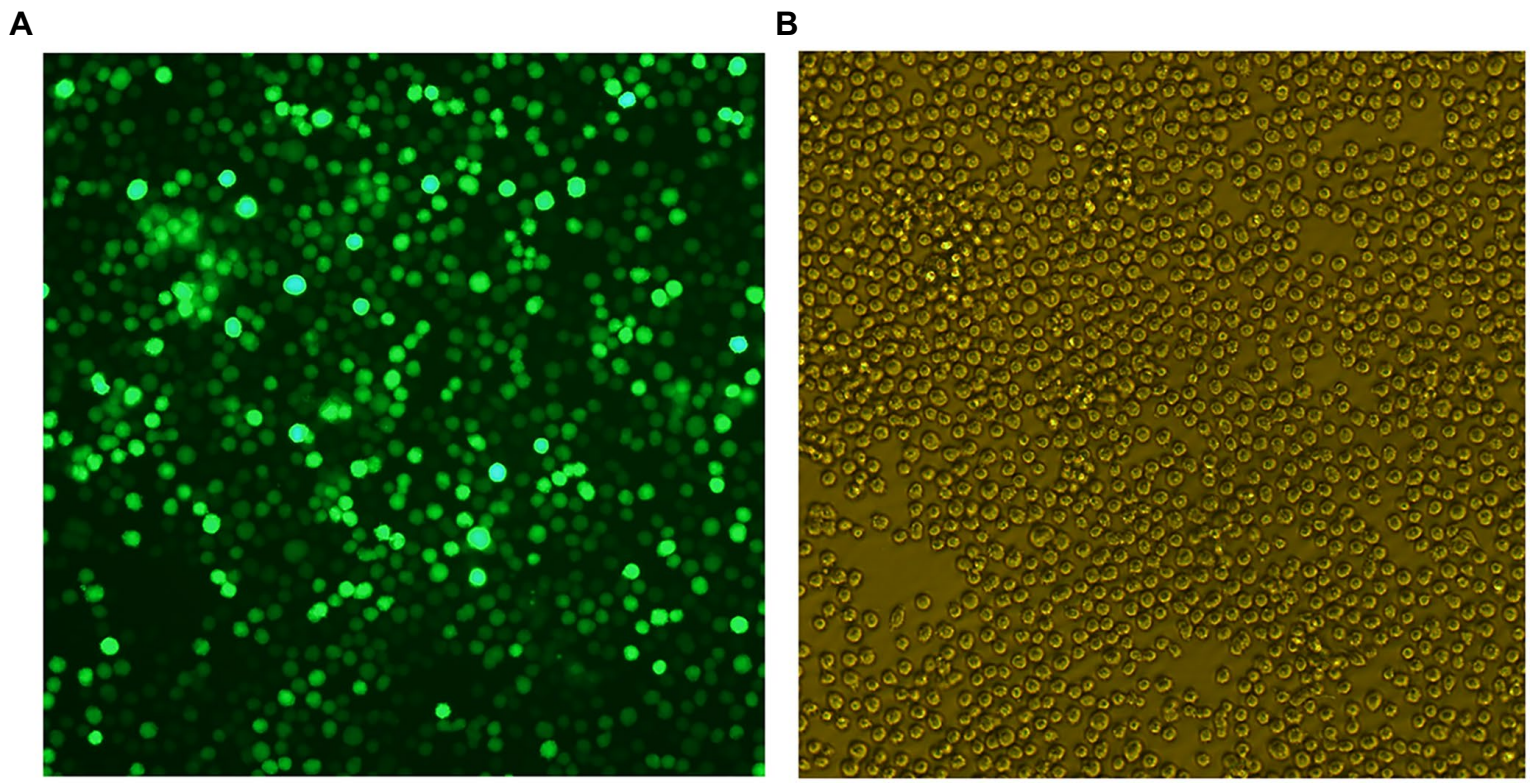
The fluorescence **(A)** and cells **(B)** of Chinese hamster ovary (CHO)-gE cell line.

Chinese hamster ovary cells were transduced with lentivirus to gain CHO-gE. The stable CHO-gE cell line was screened by subcloned three times using the limiting dilution method, and the CHO-gE cell with high expression of gE was screened by sandwich ELISA. The fluorescence and cells of selected CHO-gE cell line were observed as shown in Figure 3.

### Expressing and Purification of gE Protein

**Figure 4:**
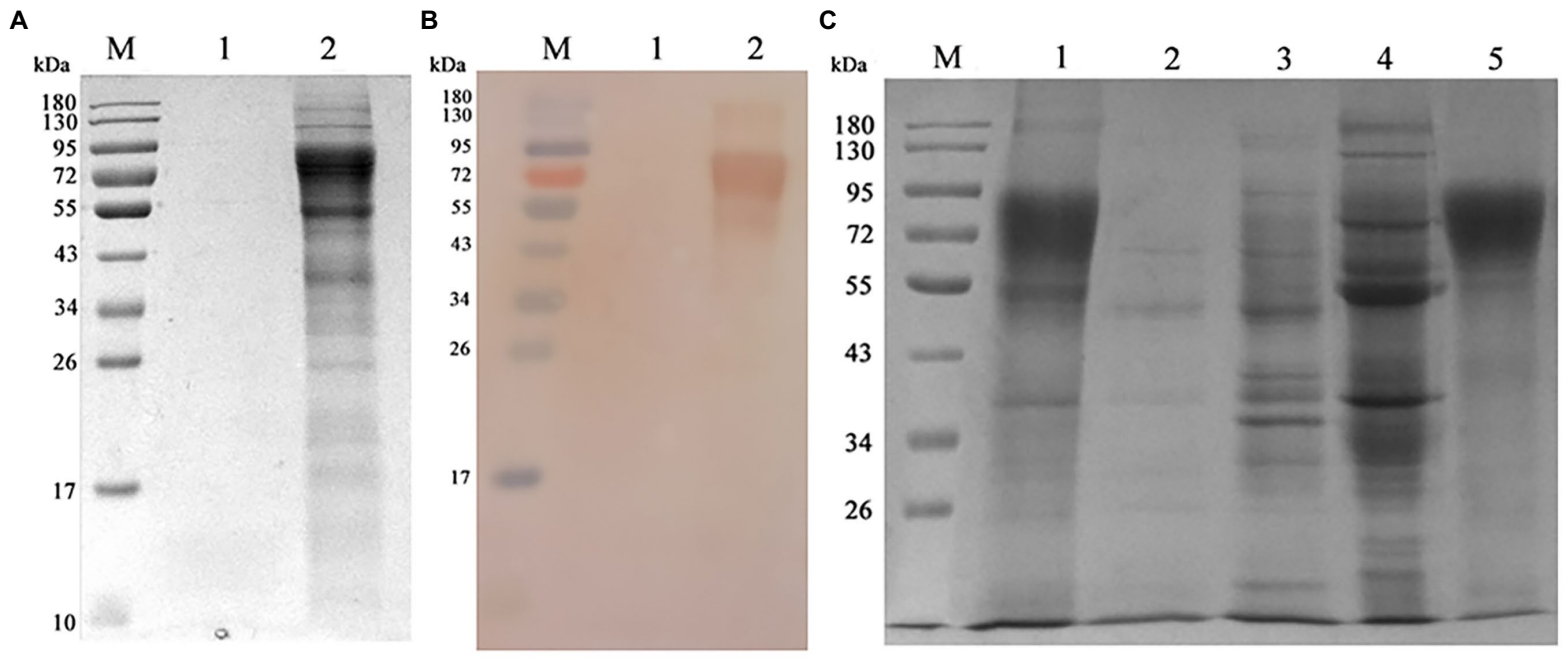
Expressing and purification of gE protein. **(A)** The CHO and CHO-gE supernatant were collected and analyzed by SDS-PAGE, separately. M: marker; 1: CHO supernatant; 2: CHO-gE supernatant. **(B)** The CHO and CHO-gE supernatant were collected and identified by Western blotting, separately. M: marker; 1: CHO supernatant; 2: CHO-gE supernatant. **(C)** The gE protein was purified by a Q-sepharose fast flow column. M: marker; 1: CHO-gE supernatant; 2: flow; 3: elution with 100 mM NaCl; 4: elution with 200 mM NaCl; and 5: elution with 300 mM NaCl.

Chinese hamster ovary-gE cells were shaking cultured, the supernatant was collected and the gE protein was purified by Q-sepharose fast flow column. As shown in Figures 4A,B, the SDS-PAGE and western blot assay showed that CHO-gE cell successfully secreted express glycosylated gE protein (70 ~ 90 kDa), which can be recognized by VZV positive serum. As shown in Figure 4C, the gE was purified by a Q-Sepharose® Fast Flow column equilibrated in 20 mM Tris–HCl, pH 7.5. The contaminating proteins were eluted from the column with the same binding buffer supplemented with 200 mM NaCl, and the gE protein was eluted by further increasing the NaCl concentration to 300 mM. The concentration of purified gE was 1 mg.ml^−1^ measured by a BCA protein assay kit and the expression quantity was 400 mg.L^−1^.

### Detection of Clinical Serum Samples

The optimal conditions and repeatability of indirect ELISA were evaluated as shown in Supplementary Tables S1–S3. Then 66 clinical samples were analyzed for the presence of VZV antibodies using the indirect ELISA kit developed in our study and commercial varicella-zoster virus IgG ELISA kit. The results (shown in Table 1) showed that the coincidence rate was 93.9% between the indirect ELISA kit and commercial ELISA kit.

**Table 1:** Detection of clinical serum samples.

Group	Indirect ELISA kit			Summary
	Result	Positive	Negative	
Commercial ELISA kit	Positive	48	4	52
	Negative	0	14	14
Summary		48	18	66

## Discussion

Varicella-zoster virus is a ubiquitous neurotropic human *alpha-herpesvirus*. It can cause varicella and HZ. During HZ, VZV multiplies and spreads within the nerve ganglia, resulted in intense inflammation, neuronal necrosis, and severe PHN. The incidence and severity of HZ and PHN increase with age, which is of particular concern in the case of aging society.

Currently, there are several vaccines have been approved for the prevention of varicella and HZ. Clinical data have revealed that the varicella vaccine can reduce the incidence of varicella by above 80% (Seward et al., 2002), with corresponding reductions of hospitalizations by 88% (Zhou et al., 2005) and deaths by 66% (Nguyen et al., 2005). The use of the live attenuated zoster vaccine markedly reduced the burden of illness due to HZ by 61.1%, the incidence of PHN by 66.5%, and the incidence of HZ by 51.3% in adults (over age 60 years; Oxman et al., 2005). A heat-treated zoster vaccine has been assessed and was shown to be generally safe and immunogenic in immunocompromised adults (Mullane et al., 2013). A subunit vaccine containing glycoprotein E combined with Adjuvant System AS01_B_ has been approved for the prevention of HZ, and it could reduce the incidence of HZ by 97.2% in adults (over age 50 years; Dendouga et al., 2012; Liu et al., 2015). Current subunit vaccines and the inactivated vaccines might be potentially safer vaccines than the live attenuated vaccines.

The gE protein is considered the major protective antigen and can stimulate both humoral and cellular immunity (Dendouga et al., 2012; Kleemann et al., 2012). The mammalian cell expression system can guide the proper protein folding, assembly, and post-translational modification (mainly glycosylated forms; Chevallier et al., 2020). In the present study, CHO cells were used to express VZV glycoprotein E ectodomain with higher expression level (400 mg.L^−1^) than previous baculovirus system (Kimura, Straus, and Williams, 1998; Liu et al., 2016). Moreover, VZV gE was secreted into the culture medium as soluble form and well-recognized by VZV positive serum, so that it is more likely to be a suitable diagnosis antigen for the detection of VZV antibodies. Then, we developed an indirect ELISA kit coated with gE protein for rapid, sensitive, and specific diagnosis of VZV antibodies. By contrast, the commercial ELISA kit relies on target antigen coated onto plate wells in the form of VZV lysate, an antigen source that is difficult to generate with batch-to-batch consistency from VZV-infected cells. Thus, as compared to results obtained using the commercial ELISA kit, our indirect ELISA kit was safer and simpler to produce. Therefore, the indirect ELISA kit developed in our study might can be used for the auxiliary diagnosis of VZV infection, vaccine efficacy studies, and seroepidemiological surveillance activities.

## Data Availability Statement

The original contributions presented in the study are included in the article/Supplementary Material, further inquiries can be directed to the corresponding author.

## Author Contributions

YN and AW contributed to perform the majority of experiments and draft the manuscript. JZ, HL, and YC conceived and designed research. PD, YQ, CL, and XZ analyzed data. GZ agreed to be accountable for all aspects of the work in ensuring that questions related to the accuracy or integrity of any part of the work are appropriately investigated and resolved. All authors contributed to the article and approved the submitted version.

## Funding

This work was funded by grants from the Zhengzhou Collaborative Innovation Major Project (Zhengzhou University).

## Conflict of Interest

The authors declare that the research was conducted in the absence of any commercial or financial relationships that could be construed as a potential conflict of interest.

## Publisher’s Note

All claims expressed in this article are solely those of the authors and do not necessarily represent those of their affiliated organizations, or those of the publisher, the editors and the reviewers. Any product that may be evaluated in this article, or claim that may be made by its manufacturer, is not guaranteed or endorsed by the publisher.

## Supplementary Material

The Supplementary Material for this article can be found online at: https://www.frontiersin.org/articles/10.3389/fmicb.2022.897752/full#supplementary-material
