# Supplementary material for: Development of an Indirect ELISA Kit for Rapid Detection of Varicella-Zoster Virus Antibody by Glycoprotein E
Source: Front Microbiol. 2022 Apr 27;13:897752. doi: 10.3389/fmicb.2022.897752 (PMC9093680; doi:10.3389/fmicb.2022.897752)
Supplement: Supplementary file 1 [file Table_1.DOCX]

**Supplementary materials**

**Development of an indirect ELISA kit for rapid detection of varicella-zoster virus antibody by glycoprotein E**

**Optimization of indirect ELISA**

The optimal concentration of coating antigen and dilution of serum sample were determined by checkerboard. The OD_450nm_ values were recorded shown in Table S1. When the concentration of coating antigen was 2 μg.ml^-1^ and the dilution of serum was 1:100, the OD_450nm_ value of positive serum was closed to 1.0 and the maximum of P/N value (the OD_450nm_ value of positive serum/ OD_450nm_ value of negative serum) appeared. Subsequently, the optimal concentration of HRP-conjugated goat anti-human IgG was further evaluated. As shown in Table S2, when the concentration of HRP-conjugated goat anti-human IgG was 1:5000, the OD_450nm_ value of positive serum was closed to 1.0 and the P/N value reached the maximum. Therefore, the optimal conditions of indirect ELISA were as follows: 2 μg.ml^-1^ of coating antigen, 1:100 dilution of serum sample and 1:5000 dilution of HRP-conjugated anti-human IgG. The cut-off value was determined by detecting negative serum samples (n = 8). The mean OD_450nm_ value was 0.184 and the standard deviation (SD) was 0.034. The cut-off value was calculated by the fomular:

cut-off value = mean OD_450nm_ value + 3 × SD

Therefore, the cut-off value was set to 0.286.

Table S1 Optimization for concentration of coating antigen and dilution of serum by square matrix titrimetry (n=3)^a^

| concentration of antigen (μg.ml^-1^) | dilution of positive serum | | | | | | |  | dilution of negative serum | | | | | | |
| --- | --- | --- | --- | --- | --- | --- | --- | --- | --- | --- | --- | --- | --- | --- | --- |
|  | 1:25 | 1:50 | 1:100 | 1:200 | 1:400 | 1:800 | 1:1600 |  | 1:25 | 1:50 | 1:100 | 1:200 | 1:400 | 1:800 | 1:1600 |
| 4 | 1.58 | 1.33 | 0.95 | 0.59 | 0.38 | 0.23 | 0.14 |  | 0.37 | 0.27 | 0.21 | 0.14 | 0.12 | 0.08 | 0.07 |
| 2 | 1.52 | 1.38 | 0.98 | 0.62 | 0.38 | 0.25 | 0.14 |  | 0.39 | 0.28 | 0.19 | 0.16 | 0.11 | 0.09 | 0.07 |
| 1 | 1.52 | 1.33 | 0.92 | 0.59 | 0.36 | 0.24 | 0.15 |  | 0.40 | 0.26 | 0.19 | 0.14 | 0.10 | 0.08 | 0.07 |
| 0.5 | 1.28 | 1.16 | 0.81 | 0.5 | 0.35 | 0.23 | 0.13 |  | 0.34 | 0.25 | 0.18 | 0.14 | 0.09 | 0.07 | 0.06 |
| 0.25 | 1.11 | 0.94 | 0.63 | 0.4 | 0.28 | 0.19 | 0.12 |  | 0.31 | 0.22 | 0.15 | 0.11 | 0.09 | 0.07 | 0.07 |
| 0.125 | 0.88 | 0.74 | 0.5 | 0.3 | 0.21 | 0.16 | 0.09 |  | 0.27 | 0.20 | 0.15 | 0.12 | 0.08 | 0.07 | 0.06 |

a. Each data was the mean of three replicates. All the SD values were < 0.07.

Table S2 Optimization for concentration of HRP-conjugated anti-human IgG (n=3)

| dilution of HRP-conjugated anti-human IgG | positive serum ^a^ | negative serum ^a^ | P/N |
| --- | --- | --- | --- |
| 1:2500 | 1.32 | 0.28 | 4.71 |
| 1:5000 | 0.96 | 0.19 | 5.05 |
| 1:10000 | 0.52 | 0.12 | 4.33 |
| 1:20000 | 0.28 | 0.08 | 3.50 |
| 1:40000 | 0.17 | 0.07 | 2.43 |
| 1:80000 | 0.12 | 0.06 | 2.00 |

a. Each data was the mean of three replicates. All the SD values were < 0.064.

**The repeatability test of indirect ELISA**

To evaluate the repeatability of indirect ELISA, 96-well plates (coated with 2 μg.ml^-1^ gE) from the same batch and three different batches were prepared to detect the serum samples (n = 10), respectively. The coefficient of variation (CV) was calculated by the fomular:

CV = SD / Mean × 100 %

As shown in Table S3, the CV of intra-assay was < 10 % and inter-assay was < 15 %, which indicated that the indirect ELISA has a good repeatability.

Table S3 The repeatability test of indirect ELISA (n=3) ^a^

| Sample number | Intra-assay repeatability | |  | Inter-assay repeatability | |
| --- | --- | --- | --- | --- | --- |
|  | Mean±SD | CV |  | Mean±SD | CV |
| 1 | 1.1134 ± 0.0159 | 1.43% |  | 1.2733 ± 0.1003 | 7.88% |
| 2 | 1.8208 ± 0.0597 | 3.28% |  | 1.7780 ± 0.1470 | 8.27% |
| 3 | 1.3601 ± 0.0670 | 4.93% |  | 1.3962 ± 0.0448 | 3.21% |
| 4 | 0.4281 ± 0.0114 | 2.67% |  | 0.4783 ± 0.0187 | 3.92% |
| 5 | 0.2482 ± 0.0115 | 4.62% |  | 0.2273 ± 0.0230 | 10.13% |
| 6 | 0.9563 ± 0.0092 | 0.97% |  | 1.0815 ± 0.0946 | 8.75% |
| 7 | 0.8671 ± 0.0538 | 6.21% |  | 0.7644 ± 0.0787 | 10.30% |
| 8 | 0.5377 ± 0.0495 | 9.21% |  | 0.6215 ± 0.0622 | 10.01% |
| 9 | 0.2446 ± 0.0221 | 9.04% |  | 0.2545 ± 0.0279 | 10.95% |
| 10 | 1.9010 ± 0.0513 | 2.70% |  | 1.9484 ± 0.0900 | 4.62% |

a. Each data was the mean of three replicates.
